# Supplementary material for: Heat adaptation of phage T7 under an extended genetic code
Source: Virus Evol. 2021 Dec 1;7(2):veab100. doi: 10.1093/ve/veab100 (PMC8923235; doi:10.1093/ve/veab100)
Supplement: veab100_Supp [file veab100_supp.zip › Sequencing_Analysis.zip!/Sequencing_Analysis/A2/evidence/SNP_4.html]

BRESEQ :: Evidence


| Predicted mutation | | | | | | |
| --- | --- | --- | --- | --- | --- | --- |
| evidence | seq id | position | mutation | annotation | gene | description |
| RA | NC\_001604.1 | 19,721 | G→A | intergenic (–/–) | – / – | –/– |

| Read alignment evidence... | | | | | | | | | | | |
| --- | --- | --- | --- | --- | --- | --- | --- | --- | --- | --- | --- |
|  | seq id | position | | ref | new | freq | score (cons/poly) | reads | annotation | genes | product |
| \* | NC\_001604.1 | 19,721 | 0 | G | A | 94.9% | 910.3 / 29.3 | 452 | intergenic (–/–) | *–/–* | –/– |
| Reads supporting (aligned to +/- strand):  **ref** base G (14/9);  **new** base A (199/227);  **total** (215/237) | | | | | | | | | | | |
| Fisher's exact test for biased strand distribution *p*-value = 2.04e-01 | | | | | | | | | | | |
| Kolmogorov-Smirnov test that lower quality scores support variant *p*-value = 9.13e-01 | | | | | | | | | | | |

Only 100 of 455 total aligned reads displayed.  

`ATTCCTAGCGAAACGCTTTAAGTACCTTGCGGAACTCCACGATTGCGATTGGGTGTCTCGTTGTAAGCATGAAGGCGAGACAATGCGTGTATACTTTAAGGAGGTATAAGTTATGGGTAAGAAAGTTAAGAAGGCCGTGAAGAAAGTCACCAAGTCCGTTAAGAAAGTCGTTAAGGAAGGGGCTCGTCCGGTTAAACAGGTTGCTGGCGGTCTAGCTGGTCTGGCTGGTGGTACTGGTGAAGCACAGATGGTGGAAGTACCACAAGCTG‑CCGCACAGATTGTTGACGTACCTGAGAAAGAGGTTTCCACTGAGGACGAAGCACAGACAGAAAGCGGACGCAAGAAAGCTCGTGCTGGCGGTAAGAAATCCTTGAGTGTAGCCCGTAGCTCCGGTGGCGGTATCAACATTTAATCAGGAGGTTATCGTGGAAGACTGCATTGAATGGACCGGAGGTGTCAACTCTAAGGGTTATGGTCGTAAGTGGGTTAATGGTAAACTTGTGACTCCACATAGGCACATCTATGAGGAGACATATGGTCCAGTTCCAACAGGAATTGTGGTGATGCATATCTGCGATAACCCTAGGTGCTATAAC  >  NC_001604.1/19423‑20018`  

                                                                                                                                                                                                                                                                                                           `|`                                                                                                                                                                                                                                                                                                           
              `gCTTTAAGTACCTTGCGGAACTCCACGATTGCGATTGGGTGTCTCGTTGTAAGCATGAAGGCGAGACAATGCGTGTATACTTTAAGGAGGTATAAGTTATGGGTAAGAAAGTTAAGAAGGCCGTGAAGAAAGTCACCCAGTCCGTTAAGAAAGTTGTTAAGGAAGGGGCTCGTCCGGTTAAACAGGTTGCTGGCGGTCTAGCTGGTCTGGCTGGTGGTACTGGTGAAGCACAGATGGTGGAAGTACCACAATCTG‑CCGCACAGATTGTTGACGTACCTGAGAAAAAGGTTTCCACTGAGGa                                                                                                                                                                                                                                                                                           >  1:329990/1‑301 (MQ=255)`  
                          `ttGCGGAACTCCACGATTGCGATTGGGTGTCTCGTTGTAAGCATGAAGGCGAGACAATGCGTGTATACTTTAAGGAGGAATAAGTTATGGGTAAGAAAGTTAAGAAGGCCGTGAAGAAAGTCACCAAGTCCGTTAAGAAAGTCGTTAAGGAAGGGGCTCTTCCGGTTAAACAGGTTGCTGGCGGTCTAGCTGGTCTGGCTGGTGGTACTTGTGAAGCACAGATGGTGGAAGTATCACAAGCTG‑CCGCACAGATTGTTGACGTACCTGAGAAAAAGGTTTCCACTGAGGACGAAGCacagac                                                                                                                                                                                                                                                                               <  1:496878/301‑1 (MQ=255)`  
                             `cGGAACTCCACGATTGCGAGTGGGTGTCTCGTTGTAAGCATGAAGGCGAGACAATGCGTGTATACTTTAAGGAGGTATAAGTTATGGGTAAGAAAGTTAAGAAGGCCGTGAAGAAAGTCACCAAGTCCGTTAAGAAAGTCGTTAAGGAAGGGGCTCGTCCGGTTAAACAGGTTGCTGGCGGTCTAGCTGGTCTGGCTGGTGGTACTGGTGAAGCACAGATGGTGGAAGTACCACAAGCTG‑CCGCACAGATTGTTGACGTACCTGAGAAAGAGGTTTCCACTGAGGACGAAGCAcagacaga                                                                                                                                                                                                                                                                            <  1:87568/301‑1 (MQ=255)`  
                                            `gctattgGGTGTCTCGTTGTAAGCATGAAGGCGAGACAATGCGTGTATAATTTAAGGAGGTATAAGTTATGGGTAAGAAAGTTAAGAAGGCCGTGAAGAAAGTCACCAAGTCCGTTAAGAAAGTCGTTAAGGAAGGGACTCGTCCGGTTAAACAGGTTGCTGGCGGTCTAGCTGGTCTGGCTGGTGGTACTGGTGAAGCACAGATGGTGGAAGTACAACAAGCTG‑CCGCACAGATTGTTGACGTACCTGAGAAAAAGGTTTCCACTGAGGACGAAGCACAGACAGAAAGCGGACGCAAGaa                                                                                                                                                                                                                                                             >  1:338065/4‑301 (MQ=255)`  
                                               `attgGGTGTCTCGTTGTAAGCATGAAGGCGAGACAATGCGTGTATACTTTAAGGAGGTATAAGTTATGGGTAAGAAAGTTAAGAAGGCCGTGAAGAAAGTCACCAAGTCCGTTAAGAAAGTCGTTAAGGAAGGTGCTCGTCCGGTTAAACAGGTTGCTGGCGGTCTAGCTGGTCTGGCTGGTGGTACTGGTGAAGCACAGATGGTGGAAGTACCACAAGCTG‑CCGCACAGATTGTTGACGTACCTTAGAAAAAGGTTTCCACTGAGGACGAAGCACAGACAGAAAGCGGACGCAAGAAAGc                                                                                                                                                                                                                                                          <  1:278964/301‑1 (MQ=255)`  
                                               `attgGGTGTCTCGTTGTAAGCATGAAGGCGAGACAATGCGTGTATACTTTAAGGAGGTATAAGTTATGGGTAAGAAAGTTAAGAAGGCCGTGAAGAAAGTCACCAAGTCCGTTAAGAAAGTCGTTAAGGAAGGGGCTCGTCCGGTTAAACAGGTTGCTGGCGGTCTAGCTGGTCTGGCTGGTGGTACTGGTGAAGCACAGATGGTGGAAGTACCACAAGCTG‑CCGCACAGATTGTTGACGTACCTGAGAAAAAGGTTTCCACTGAGGACGAAGCACAGACAGAAAGCGGACGCAAGAAAGc                                                                                                                                                                                                                                                          <  1:650835/301‑1 (MQ=255)`  
                                                     `tgtCTCGTTGTAAGCATGAAGGCGAGACAATGCGTGTATACTTTAAGGAGGTATAAGTTATGGGTAAGAAAGTTAAGAAGGCCGTGAAGAAAGTCACCAAGTCTGTTAAGAAAGTCGTTAAGGAAGGGGCTCGTCCGGTTAAACAGGTTGCTGGCGGTCTAGCTGGTCTGGCTGGTGGTACTGGTGAAGCACAGATGGTGGAAGTACCACAAGCTG‑CCGCACAGATTGTTGACGTACCTGAGAAAAAGGTTTCCACTGAGGACGAAGCACAGACAGAAAGCGGACGCAAGAAAGCTCGTGc                                                                                                                                                                                                                                                    <  1:372442/301‑1 (MQ=255)`  
                                                                        `gggCGAGACAATGCGTGTATACTTTAAGTAGGGATAAGTTATGGGTAAGAAAGTTAAGAAGGCCGTGAAGAAAGTCACCAAGTCCGTTAAGAAAGTCGTTAAGGAAGGGGCTCGTCCGGTTAAACAGGTTGCTGGCGGTCTAGCTGGTCTGGCTGGTGGTACTGGTGAAGCACAGATGGTGGAAGTACCACAAGCTG‑CCGCACAGATTGTTGACGTACCTGAGAAAAAGGTTTCCACTGAGGACGAAGCACAGACATAAAGCGGATGCAAGAAAGCTCGTGCTGGCGGTAAGAAATCCTTg                                                                                                                                                                                                                                 <  1:770502/300‑1 (MQ=255)`  
                                                                        `aGGCGAGACAATGCGTGTATACTTTAAGGAGGTATAAGTTATGGGTAAGAAAGTTAAGAAGGCCGTGAAGAAAGTCACCAAGTCCGTTAAGAAAGTCGTTAAGGAAGGGGCTCGTCCGGTTAAACAGGTTGCTGGCGGTCTAGCTGGTCTGGCTGGTGGTACTGGTGAAGCACAGATGGTGGAAGTACCACAAGCTG‑CCGCACAGATTGTTGACGTACCTGAGAAAAAGGTTTCCACTGAGGACGAAGCACAGACAGAAAGCGGACGCAAGAAAGCTTGTGCTGGCGGTAAGAAATCCTTg                                                                                                                                                                                                                                 >  1:482689/1‑301 (MQ=255)`  
                                                                           `ggagaCAATGCGTGTATACTGTGAGGAGGTAAAAGTTAAGGGAAAGAAAGTTAAGAAGGCCGTGAAGAAAGTCACCAAGTCCGTTAAGAAAGTCGGTAAGGAAGGGGCTCGTCCGGTTAAACAGGTAGCTGGCGGTCTAGCTGGTCTGTCTGGTGGTACTGGTGAAGCACAGATGGTGGAAGTATCACAAGCTG‑CCGCACAGATTGTTGACGTACCTGAGAAAAAGGTTTCCACTGAGGACGAAGCACAGACAGAAAGCGGACGCAAGAAAGCTCGTGCTTGCGGTAAGAAATCCTTGAgt                                                                                                                                                                                                                              <  1:125894/300‑1 (MQ=255)`  
                                                                            `gagaCAATGCGTGTATACTTTAATGAGGTATAAGTTATGGGTAAGAAAGTTAAGAAGGCCGTGAAGAAAGTCACCAAGTCCGTTAAGAAAGTCGTTAAGGAAGGGGCTCGTCCGGTTAAACAGGTTGCTGGCGGTCTAGCTGGTCTGGCTGGTGGTACTTGTGAAGCACAGATGGTGGAAGTACCACAAGCTG‑CCGCACAGATTGTTGACGTACCTGAGAAAAAGGTTTCCACTTAGGACGAAGCACAGACAGAAAGCGGACGCAAGAAAGCTCGTGCTGGCGGTAAGAAATCCTTGAgtg                                                                                                                                                                                                                             <  1:689490/301‑1 (MQ=255)`  
                                                                                 `ggagatgtgtataagagacagggTATAAGTTATGGGTAAGAAAGTTAAGAAGGCCGTGAAGAAAGTCACCAAGTCCGTTAAGAAAGTCGTTAAGGAAGGGGCTCGTCCGGTTAAACAGGTTGCTGGCGGTCTAGCTGGTCTGGCTGGTGGTACTGGTGAAGCACAGATGGTGGAAGTACCACAATCTG‑CCGCACAGATTGTTGATGTACCTGAGAAAAAGGTTTCCACTGAGGACGAAGCACAGACAGAAAGCGGACGCAAGAAAGCTCGTGCTGGCGGTAAGAAATCCTTGAGTGTAGcc                                                                                                                                                                                                                        <  1:21964/280‑1 (MQ=255)`  
                                                                                    `gCGTGTATACTTTAAGGAGGTATAAGTTATGGGTAAGAAAGTTAAGAAGGCTGTGAAGAAAGTCACCAAGTCCGTTAAGAAAGTCGTTAAGGAAGGGGCTCGTCCGGTTAAACAGGTTGCTGGCGGTCTAGCTGGTCTGGCTGGTGGTACTGGTGAAGCACAGATGGTGGAAGTACCACAAGCTG‑CCGCTCAGATTGTTGACGTACCTGAGAAAAAGGTTTCCACTTAGGACGAAGCACAGACAGAAAGCGGACGCAAGAAAGCTCGTGCTGGCGGTAAGAAATCCTTGAGTGTAGCCAGt                                                                                                                                                                                                                     >  1:814714/1‑301 (MQ=255)`  
                                                                                         `ataagagacaggaggTATAAGTTATTGGTAAGAAAGTTAAGAAGGCCGTGAAGAAAGTCACCAAGTCCGTTAAGAAAGTCGTTAAGGAAGGGGCTCGTCCGGTTAAACAGGTTGCTGGCGGTCTAGCTGGTCTGGCTGGTGGAACTGGTGAAGCACAGATGGTGGAAGTACAACAAGCTG‑CCGCACAGATTGTTGACGTACCTGAGAAAAAGGTTTCCACTGAGGACGAAGCACAGACAGAAAGCGGACGCAAGAAAGCTCGTGCTGGCGGTAAGAAATCCTTGAGTGTAGCCCGTAGCTc                                                                                                                                                                                                                <  1:458616/292‑1 (MQ=255)`  
                                                                                             `cTTTAAGGAGGTATAAGTTATGGGTAAGAAAGTTAAGAAGGCCGTGAAGAAAGTCACCAAGTCCGTTAAGAAAGTCGTTAAGGAAGGGGCTCGTCCGGTTAAACAGGTTGCTGGCGGTCTAGCTGGTCTGGCTGGTGGTACTGGTGAAGCACAGATGGTGGAAGTACCACAAGCTG‑CCGCACAGATTGTTGACGTACCTGAGAAAAAGGTTTCCACTGAGGACGAAGCACAGACAGAAAGCGGACGCAAGAAAGCTCGTGCTGGCGGTAAGAAATCCTTGAGTGTAGCCCGTAGCTCCggg                                                                                                                                                                                                            >  1:661296/1‑300 (MQ=255)`  
                                                                                                   `gaaggTATAAGTTATGGGTAAGAAAGTTAAGAAGGCAGTGAAGAAAGTCACCAAGTCCGTTAAGAAAGTCGTTAAGGAAGGGGCTCGTCCGGTTAAACAGGTTGCTGGCGGTCTAGCTGGTCTGGCTGGTGGTACTGGTGAAGCACAGATGGTGGAAGTACCACAAGCTG‑CCGCACAGATTGTTGACGTACCTGAGAAAAAGGTTTCCACTGAGGACGAAGCACAGACAGAAAGCGGACGCAAGAAAGCTCGTGCTGGCGGTAAGAAATCCTTGAGTGTAGCCCGTAGCTCAGGTGGCGGt                                                                                                                                                                                                      <  1:417494/299‑1 (MQ=255)`  
                                                                                                      `ggTAGAAGTTATGGGAAAGAAAGTTAAGAAGGCCGTGAAGAAAGTCACCAAGTCCGTTAAGAAAGTCGTTAAGGAAGGGGCTCGTCCGGTTAAACAGGTTGCTGGCGGTCTAGCTGGTCTGGCTGGTGGTACTGGTGAAGCACAGATGGTGGAAGTACCACAAGCTG‑CCGCACAGATTGTTGACGTACCTGAGAAAAAGGTTTCCACTGAGGACGAAGCACAGACAGAAAGCGGACGCAAGAAAGCTCGTGCTGGCGGTAAGAAATCCTTGAGTGTAGCCCGTAGCTCCGGTGGCGGTATc                                                                                                                                                                                                   <  1:223143/301‑1 (MQ=255)`  
                                                                                                           `aaGTTATGGGTAAGAAAGTTAAGAAGGCCGTGAAGAAAGTCACCAAGTCCGTTAAGAAAGTCGTTAAGGAAGGGGCTCGTCCGGTTAAACAGGTTGCTGGCGGTCTAGCTGGTCTGGCTGGTGGTACTGGTGAAGCACAGATGGTGGAAGTACCACAAGCTG‑CCGCACAGATTGTTGACGTACCTGAGAAAAAGGTTTCCACTGAGGACGAAGCACAGACAGAAAGCGGACGCAAGAAAGCTCGTGCTGGCGGTAAGAAATCCTTGAGTGTAGCCCGTAGCTCCGGTGGCGGTATCAACAt                                                                                                                                                                                              <  1:365187/301‑1 (MQ=255)`  
                                                                                                             `gTTATGGGTAAGAAAGTTAAGAAGGCTGTGAAGAAAGTCACCAAGTCCGTTAAGAAAGTCGTTAAGGAAGGGGCTCGTCCGGTTAAACAGGTTGCTGGCGGTCTAGCTGGTCTGGCTGGTGGTACTGGTGAAGCACAGATGGTGGAAGTACCACAAGCTG‑CCGCACAGATTGTTGACGTACCTGAGAAAAAGGTTTCCACTGAGGACGAAGCACAGACAGAAAGCGGACGCAAGAAAGCTCGTGCTGGCGGTAAGAAATCCTTGAGTGTAGcctctgtctcttatacacatctccgagccc                                                                                                                                                                                            >  1:505688/1‑273 (MQ=255)`  
                                                                                                                 `ggggctcggagatgtgtataagagacaggaaAGTCACCAAGTCCGTTAAGAAAGTCGTTAAGGAAGGGGCTCGTCCGGTTAAACAGGTTGCTGGCGGTCTAGCTGGTCTGGCTGGTGGTACTGGTGAAGCACAGATGGTGGAAGTACCACAAGCTG‑CCGCACAGATTGTTGACGTACCTGAGAAAAAGGTTTCCACTGAGGACGAAGCACAGACAGAAAGCGGACGCAAGAAAGCTCGTGCTGGCGGTAAGAAATCCTTGAGTGTAGCCCGTAGCTCCGGTGGCGGTATCAACATTTAATc                                                                                                                                                                                        <  1:205042/273‑1 (MQ=255)`  
                                                                                                                          `aaGTTAAGAAGGCCGTGAAGAAAGTCACCAAGTCCGTTAAGAAAGTCGTTAAGGAAGGGGCTCGTCCGGTTAAACAGGTTGCTGGCGGTCTAGCTGGTCTGGCTGGTGGTACTGGTGAAGCACAGATGGTGGAAGTACCACAAGCTG‑CCGCACAGATTGTTGACGTACCTGAGAAAAAGGTTTCCACTGAGGACGAAGCACAGACAGAAAGCGGACGCAAGAAAGCTCGTGCTGGCGGTAAGAAATCCTTGAGTGTAGCCCGTAGCTCCGGTGGCGGTATCAACATTTAATCAGGAGGTTa                                                                                                                                                                               >  1:239699/1‑301 (MQ=255)`  
                                                                                                                            `gTTAAGAAGGCCGTGAAGAAAGTCACCAAGTCCGTTAAGAAAGTCGTTAAGGAAGGGGCTCGTCCGGTTAAACAGGTTGCTGGCGGTCTAGCTGGTCTGGCTGGTGGTACTGGTGAAGCACAGATGGTGGAAGTACCACAAGCTG‑CCGCACAGATTGTTGACGTACCTGAGAAAAAGGTTTCCACTGAGGACGAAGCACAGACAGAAAGCGGACGCAAGAAAGCTCGTGCTGGCGGTAAGAAATCCTTGAGTGTAGCCCGTAGCTCCGGTGGCGGTATCAACATTTAATctgtctcttata                                                                                                                                                                             >  1:709048/1‑290 (MQ=255)`  
                                                                                                                                     `gCCGTGAAGAAAGTCACCAAGTCCGTTAAGAAAGTCGTTAAGGAAGGGGCTCGTCCGGTTAAACAGGTTGCTGGCGGTCTAGCTGGTATGGCTGGTGGTACTGGTGAAGCACAGATGGTGGAAGTACCACAAGCTG‑CCGCACAGATTGTTGACGTACCTGAGAAAACGGTTTCCACTGAGGACGAAGCACAGACAGAAAGCGGACGCAAGAAAGCTCGTGCTGGCGGTAAGAAATCCTTGCGTGTAGCCCGTAACTCCGGTTGCGGTATCAACATTTAATCCGGAGGTTCTCATGGAAGAc                                                                                                                                                                    >  1:812321/1‑301 (MQ=255)`  
                                                                                                                                          `gaagaaAGTCACCAAGTCCGTTAAGAAAGTCGTTAAGGAAGGGGCTCGTCCGGTTAAACAGGTTGCTGGCGGTCTAGCTGGTCTGGCTGGTGGTACTGGTGAAGCACAGATGGTGGAAGTACCACAAGCTG‑CCGCACAGATTGTTGACGTACCTGAGAAAAAGGTTTCCACTGAGGACGAAGCACAGACAGAAAGCGGACGCAAGAAAGCTCGTGCTGGCGGTAAGAAATCCTTGAGTGTAGCCCATAGCTCCGGTGGCGGTATCAACATTTAATCAGGAGGTTATCGTGGAAGACTGCAt                                                                                                                                                               <  1:555935/301‑1 (MQ=255)`  
                                                                                                                                          `gaagaaAGTCACCAAGTCCGTGAAGAAAGTCGTTAAGGAAGGGGCTCGTCCTGTTAAACAGGTTGCTGGCGGTCTAGCTGGTCTGGCTGGTGGTACTGGTGAAGCACAGATGGTGGAAGTACCACAAGCTG‑CCGCACAGATTGTTGACGTACCTGAGAAAAAGGTTTCCACTGAGGACGAAGCACAGACAGAAAGCGGACGCAAGAAAGCTCGTGCTGGCGGTAAGAAATCCTTGAGTGTAGCCCGTAGCTCCGGTGGCGGTATCAACATTTAATCAGGAGGTTATCGTGTAAGACTGCAt                                                                                                                                                               <  1:523337/301‑1 (MQ=255)`  
                                                                                                                                               `aaGTCACCAAGTCCGTTAAGAAAGTCGTTAAGGAAGGGGCTCGTCTGGTTAAACAGGTTGCTGGCGGTCTAGCTGGTCTGGCTGGTGGTACTGGTGAAGCACAGATGGTGGAAGTACCACAAGCTG‑CCGCACAGATTGTTGACGTACCTGAGAAAAAGGTTTCCACTGAGGACGAAGCACAGACAGAAAGCGGACGCAAGAAAGCTCGTGCTGGCGGTAAGAAATCCTTGAGTGTAGCCCGTAGCTCCGGTGGCGGTATCAACATTTAATCAGGAGGTTATCGTGGAAGACTGCATTGAAt                                                                                                                                                          <  1:796928/301‑1 (MQ=255)`  
                                                                                                                                               `aaGTCACCAAGTCCGTTAAGAAAGTCGTTAAGGAAGGGGCTCGTCCGGTTAAACAGGTTGCTGGCGGTCTAGCTGGTCTGGCTGGTGGTACTGGTGAAGCACAGATGGTGGAAGTACCACAAGCTG‑CCGCACAGATTGTTGACGTACCTGAGAAAAAGGTTTCCACTGAGGACGAAGCACAGACAGAAAGCGGACGCAAGAAAGCTCGTGCTGGCGGTAAGAAATCCTTGAGTGTAGCCCGTAGCTCCGGTGGCGGTATCAACATTTAATCAGGAGGTTATCGTGGAAGACTGCATTGAAt                                                                                                                                                          <  1:356419/301‑1 (MQ=255)`  
                                                                                                                                               `aaGTCACCAAGTCCGTTAAGAAAGTCGTTAAGGAAGGGGCTCGTCCGGTTAAACAGGTTGCTGGCGGTCTAGCTGGTCTGGCTGGTGGTACTGGTGAAGCACAGATGGTGGAAGTACCACAAGCTG‑CCGCACAGATTGTTGACGTACCTGAGAAAAAGGTTTCCACTGAGGACGAAGCACAGACAGAAAGCGGACGCAAGAAAGCTCGTGCTGGCGGTAAGAAATCCTTGAGTGTAGCCCGTAGCTCCGGTGGCGGTATCAACATTTAATCAGGAGGTTATCGTGGAAGACTGCATTGAAt                                                                                                                                                          <  1:103440/301‑1 (MQ=255)`  
                                                                                                                                               `aaGGCACCAAGTCCGTTAAGAAAGTCGTTAAGGAAGGGGCTCGTCAGGTTAAACAGGTTGCTGGCGGTCTAGCTGGTCTGGCTGGTGGTACTGGTGAAGCACAGATGGTGGAAGTACCACAAGCTG‑CCGCACAGATTGTTGACGTACCTGAGAAAAAGGTTTCCACTGAGGACGAAGCACAGACAGAAAGCGGACGCAAGAAAGGTCGTGCTGGCGGTAAGAAATCCTTGAGTGTAGCGCGTAGCTCCGGTGGCAGTATCAACATTTAATCAGGGGGTTATCGTGGAAGACTGCATTGAAt                                                                                                                                                          <  1:114734/301‑1 (MQ=255)`  
                                                                                                                                                 `gTCACCAAGTCCGTTAAGAAAGTCGTTAAGGAAGGGGCTCGTCCGGTTAAACAGGTTGCTGGCGGTCTAGCTGGTCTTGCTGGTGGTACTGGTGAAGCACAGATGGTGGAAGTACCACAAGCTG‑CCGCACAGATTGTTGACGTACCTGAGAAAAAGGTTTCCACTGAGGACGAAGCACAGACAGAAAGCGGACGCAAGAAAGCTCGTGCTGGCGGTAAGAAATCCTTGAGTGTAGCCCGTAGCTCCGGTGGCGGTATCAACATTTAATCAGGAGGTTATCGTGGAAGACTGCATTGAATgg                                                                                                                                                        >  1:453808/1‑301 (MQ=255)`  
                                                                                                                                                 `gTCACCAAGTCCGTTAAGAAAGTCGTTAAGGAAGGGGCTCGTCCGGTTAAACAGGTTGCTGGCGGTCTAGCTGGTCTGGCTGGTGGTACTGGTGAAGCACAGATGGTGGAAGTACCACAAGCTG‑CCGCACAGATTGTTGACGTACCTGAGAAAAAGGTTTCCACTGAGGACGAAGCACAGACAGAAAGCGGACGCAAGAAAGCTCGTGCTGGCGGTAAGAAATCCTTGAGTGTAGCCCTTAGCTCCGGTGGCGGTATCAACATTTAATCAGGAGGTTATCGTGGAAGACTGCATTGAAtct                                                                                                                                                        >  1:606966/1‑299 (MQ=255)`  
                                                                                                                                                 `gTCACCAAGTCCGTTAAGAAAGTCGTTAAGGAAGGGGCTCGTCCGGTTAAACAGGTTGCTGGCGGTCTAGCTGGTCTGGCTGGTGGTACTGGTGAAGCACAGATGGTGGAAGTACCACAAGCTG‑CCGCACAGATTGTTGACGTACCTGAGAAAAAGGTTTCCACTGAGGACGAAGCACAGACAGAAAGCGGACGAAAGAAAGCTCGTGCTGGCGGTAAGAAATCCTTGAGTGTAGCCCGTAGCTCCGGTGGCGGTATCAACATTTAATCCGGAGGTTATCGTGGAAGACTGCATTGAATgg                                                                                                                                                        >  1:424354/1‑301 (MQ=255)`  
                                                                                                                                                         `ggccGTTAAGAAAGTCGTTAAGGAAGTGGCTCGTCCTGTTAAACAGGTTGCTGGCGGTCTAGCTGGTCTGGCTGGTGGTACTGGTGAAGCACAGATGGTGGAAGTACCACAATCTG‑CCGAACAGATTGTTGACATACCTGAGAAAAAGGTTTCCACTGAGGACGAAGCACAGACAGAAAGCGGAAGCAAGAAAGCTCGTGCTGGCGGTAAGAAATCCTTGAGTGTAGCCCGTAGCTCCAGTTGCGGTATCAACATTTAATCAGGAGGTTATCGTGGAAGACTGCATTGAATGGACCGGAgg                                                                                                                                                <  1:806928/299‑1 (MQ=255)`  
                                                                                                                                                         `gTCCGTTAAGAAAGTCGTTAAGGAAGGGGCTCGTCCGGTTAAACAGGTTGCTGGCGGTCTAGCTGGTCTGGCTGGTGGTACTGGTGAAGCACAGATGGTGGAAGTACCACAAGCTG‑CCGCACAGATTGTTGACGTACCTGAGAAAAAGGTTTCCACTGAGGACGAAGCACAGACAGAAAGCAGACGCAAGAAAGCTCGTGCTGGCGGTAAGAAATCCTTGAGTGTAGCCCGTAGCTCCGGTGGCGGTATCAACATTTAATCAGGAGGTTATCGTGGAAGACTGCATTGAATGGACaggagg                                                                                                                                                >  1:42870/1‑301 (MQ=255)`  
                                                                                                                                                         `gTCCGTTAAGAAAGTCGTTAAGGAAGGGGCTCGTCCGGTTAAACAGGTAGCTGGCGGTCTAGCTGGTCTGGCTGGTGGTACTGGTGAAGCATAGAGGGTGGAAGTACCACAAGCTG‑CCGCACAGATTGTTGACGTACCTGAGAAAAAGGTTTCCACTGAGGACGAAGCACAGACAGAAAGTGGACGCAAGAAAGCTCGTGCTGGCGGTAAGAAATCCTTGAGTGTAGCCCGTAGCTCCGGTGGCGGTATCAACATTTAATCAGGAGGTTATCGTGGAAGACTGCATTGAATGGACCGGAgg                                                                                                                                                <  1:784086/301‑1 (MQ=255)`  
                                                                                                                                                         `cggagatgtgtataagggacaggaaggGGCTCGTCCGGTTAAACAGGTTGCTGGCGGTCTAGCTGGTCTGGCTGGTGGTACTGGTGAAGCACAGATGGTGGAAGTACCACAAGCTG‑CCGCACAGATTGTTGACGTACCTGAGAAAAAGGTTTCCACTGAGGACGAAGCACAGACAGAAAGCGGACGCAAGAAAGCTAGTGCTGGCGGTAAGAAATCCTTGAGTGTAGCCCGTAGCTCCGGTGGCGGTATCAACATTTAATCAGGAGGTTATAGTGGAAGACTGCATTGAATGGACCGGAgg                                                                                                                                                <  1:23221/281‑1 (MQ=255)`  
                                                                                                                                                                `aaGAAAGTCGTTAAGGAAGGGGCTCTTCCGGTTAAACAGGTTGCTGGCGGTCTAGCTGGTCTGGCTGGTGTTACTGGTGAAGCACAGATGGTGGAAGTACCACAAGCTG‑CCGCACAGATTGTTGACGTACCTGAGAAAAAGGTTTCCACTGAGGACGAAGCACAGACAGAAAGCGGACGCAAGAAAGCTCGTGCTGGCGGTAAGAAATCCTTGAGTGTAGCCCGTAGCTCCGGTGGCGGTATCAACATTTAATCAGGAGGTTATCGTGGAAGACTGCATTGAATGGACCGGAGGTGTCAAc                                                                                                                                         <  1:72805/301‑1 (MQ=255)`  
                                                                                                                                                                        `ggtgggctcggagatgtgtataagagacagggTTGCTGGCGGTCTAGCTGGTCTGGCTGGTGGTACTGGTGAAGCACAGATGGTGGAAGTACCACAAGCTG‑CCGCACAGATTGTTGACGTACCTGAGAAAGAGGTTTCCTCTGAGGACGAAGCACAGACAGAAAGCGGACGCAAGAAAGCTCGTGCTGGCGGTAAGAAATCCTTGAGTGTAGCCCGTAGCTCCGGTGGCGGTATCAACATTTAATCAGGAGGTTATCGTGGAAGACTGCATTGAATGGACCGGAGGTGTCAACTCTAAggg                                                                                                                                 <  1:645584/271‑1 (MQ=255)`  
                                                                                                                                                                        `gagacagggagggGCTCGTCCGGTTAGACAGGTTGCAGGCGGTATAGGTGGTCTGGCTGGTGGTACTGGGGAAGCAAAGATGGTGGAAGTAGCACAAGCTG‑CCGAACAGATTGTTGACGTACCTGAGAAAAAGGTTTCCAATGAGGACGAAGCAAAGACAGAAAGCGGAAGAAAGAAAGCTCGTGCTGGAGGTAAGAAATCCTTGAGTGTAGCCCGTAGATCCGGTGGCGGTATCAACATTTAATCAGGAGGTTATCGTGGAAGACTGCATTGAATGGACCGGAGGTGTCAACTCTAAggg                                                                                                                                 <  1:766203/296‑1 (MQ=255)`  
                                                                                                                                                                        `cgtgggctcggagatgtgtataagagacagggTTGCTGGCGGTCTAGCTGGTCTGGCTGGTGGTACTGGTGAAGCACAGATGGTGGAAGTACCACAAGCTG‑CCGCACAGATTGTTGACGTACCTGAGAAAAAGGTTTCCACTGAGGACGAAGCACAGACAGAAAGCTGACGCAAGAAAGCTCGTGCTGTCGGTAAGAAATCCTTGAGTGTAGCCCGTAGCTCCGGTGGCGGTATCAACATTTAATCAGGAGGTTATCGTGGAAGACTGCATTGAATGGACCGGAGGTGTCAACTCTAAggg                                                                                                                                 <  1:671611/271‑1 (MQ=255)`  
                                                                                                                                                                        `cGTTAAGGAAGGGGCTCGTCCGGTTAAACAGGTTGCTGGCGGTCTAGCTGGTCTGGCTGGTGGTACTGGTGAAGCACAGATGGTGGAAGTACCACAAGCTG‑CCGCACAGATTGTTGACGTACCTGAGAAAAAGGTTTCCACTGAGGACGAAGCACAGACAGAAAGCGGACGCAAGAAAGCTCGTGCTGGCTGTAAGAAATCCTTGAGTGTAGCCCGTAGCTCCCGTGGCGGTATCAACATTTAATCAGGAGGTTATCGTGGAAGACTGCATTGAATGGACCGGAGGTGTCAACTCTAAggg                                                                                                                                 <  1:682817/301‑1 (MQ=255)`  
                                                                                                                                                                        `cGTTAAGGAAGGGGCTCGTCCGGTTAAACAGGTTGCTGGCGGTCTAGCTGGTCTGGCTGGTGGTACTGGTGAAGCACAGATGGTGGAAGTACCACAAGCTG‑CCGCACAGATTGTTGACGTACCTGAGAAAAAGGTTTCCACTGAGGACGAAGCACAGACAGAAAGCGGACGCAAGAAAGCTCGTGCTGGCGGTAAGAAATCCTTGAGTGTAGCCCGTAGCTCCGGTGGCGGTATCAACATTTAATCAGGAGGTTATCGTGGAAGACTGCATTGAAtctgtctcttatacacatctccgagc                                                                                                                                 >  1:438665/1‑276 (MQ=255)`  
                                                                                                                                                                        `cGTTAAGGAAGGGGCTCGTCCGGTTAAACAGGTTGCTGGCGGTCTAGCTGGTCTGGCTGGTGGTACTGGTGAAGCACAGATGGTGGAAGTACCACAAGCTG‑CCGCACAGATTGTTGACGTACCTGAGAAAAAGGTTTCCACTGAGGACGAAGCACAGACAGAAAGCGGACGCAAGAAAGCTCGTGCTGGCGGTAAGAAATCCTTGAGTGTAGCCCGTAGCTCCGGTGGCGGTATCAACATTTAATCAGGAGGTTATCGTGGAAGACTGCATTGAATGGACCGGAGGTGTCAACTCTAAggg                                                                                                                                 >  1:389268/1‑301 (MQ=255)`  
                                                                                                                                                                          `gagatgtgtataagagacaggTTAAACAGGTTGCTGGCGGTCTAGCTGGTCTGGCTGGTGGTACTGGTGAAGCACAGATGGTGGAAGTACCACAAGCTG‑CCGCACAGATTGTTGACGTACCTGAGAAAAAGGTTTACACTGAGGACGAAGCACAGACAGAAAGCGGACGCAAGAAAGCTCGTGCTGGCGGTAAGAAATCCTTGAGTGTAGCCCGTAGCTCCGGTGGCGGTATCAACATTTAATCAGGAGGTTATCGTGGAAGACTGCATTGAATGGACCGGAGGTGTCAACTCTAAGGGtt                                                                                                                               <  1:152295/282‑1 (MQ=255)`  
                                                                                                                                                                             `aggaaggGGCTCGTCCGGTTAAACAGGTTGCTGGCGGTCTAGCTGGTCTGGCTGGTGGTACTGGTGAAGCACAGATGGTGGAAGTACCACAAGCTG‑CAGCACAGATTGTTGACGTACCTGAGAAAAAGGTTTCCACTGAGGACGAAGCACAGACAGAAAGCGGACGCAAGAAAGCTCGTGCTGGCGGTAAGAAATCCTTGAGTGTAGCCCGTAGCTCCGGTGGCGGTATCAACTTTTAATCAGGAGGTTATCGTGGAAGACTGCATTGAATGGACCGGAGGTGTCAACTATAAggctgtct                                                                                                                            >  1:514878/1‑295 (MQ=255)`  
                                                                                                                                                                              `ggaaggGGCTCGGCGGGGTGAACAGGTTGCGGGCGGTCTAGCTGGTCTGGCTGGGGGTACTGGTGAAGCACAGATGGTGGAAGTACCACAAGCTG‑CCGCACAGATTGTTGACGTACGTGAGAAAAAGGTTTCCACTGAGGACGAAGCACAGACAGAAAGCGGACGCAAGAAAGCTCGTGCTGGCGGTAAGAAATCCTTGAGTGTAGCCCGTAGCTCCGGTGGCGGTATCAACATTTAATCAGGAGGTTATCGTGGAAGACTGCATTGAATGGACCGGAGGTGTCAACTCTAAGGGTTATgg                                                                                                                           <  1:586220/301‑1 (MQ=255)`  
                                                                                                                                                                               `gaaggGGCTCGTCCGGTTAAACAGGTTGCTGGCGGTCTAGCTGGTCTGGCTGGTGGTACTGGTGAAGCACAGATGGTGGAAGTACCACAAGCTG‑CCGCACAGATTGTTGACGTACCTGAGAAAAAGGTTTCCACTGAGGACGAAGCACAGACAGAAAGCGGACGCAAGAAAGCTCGTTCTGGCGGTAAGAAATCCTTGAGTGTAGCCCGTAGCTCCGGTGGCGGTATCAACATTTAATCAGGAGGTTATCGTTGAAGACTGCATTGAATGGACCGGAGGTGTCAACTCTAAGGGTTATGGt                                                                                                                          <  1:558588/301‑1 (MQ=255)`  
                                                                                                                                                                                      `tgtgtataagagacagggTTGCTGGCGGTCTAGCTGGTCTGGCTGGTGGTACTGGTGAAGCACAGATGGTGGAAGTACCACAAGCTG‑CCGCACAGATTGTTGACGTACCTGAGAAAAAGGTTTCCACTGAGGACGAAGCACAGACAGAAAGCGGACGCAAGAAAGCTCGTGCTGGCGGTAAGAAATCCTTGAGTGTAGCCCGTAGCTCCGGTGGCGGTATCAACATTTAATCAGGAGGTTATCGTGGAAGACTGCATTGAATGGACCGGAGGTGTAAACTCTAAGGGTTATGGTCGTAAGt                                                                                                                   <  1:294391/285‑1 (MQ=255)`  
                                                                                                                                                                                                `tAAACAGGTTGCTGGCGGTCTAGCTGGTCTGGCTGGTGGTACTGGTGAAGCACAGATGGTGGAAGTACCACAAGCTG‑CCGCACAGATTGTTGACGTACCTGAGAAAAAGGTTTCCACTGAGGACGAAGCACAGACAGAAAGCGGACGCAAGAAAGCTCGTGCTGGCGGTAAGAAATCCTTGAGTGTAGCCCGTAGCTCCAGTGGCGGTATCAACATTTAATCAGGAGGTTATCGTGGAAGACTGCATTGAATGGACCGGAGGTGTCAACTCTAAGGGTTATGGTCGTAAGTGGGTTAATgg                                                                                                         <  1:120823/301‑1 (MQ=255)`  
                                                                                                                                                                                                `atgtgtataagagacaggTCTAGCTGGGCTGGCTGGTGGTACTGGTGAAGCACAGATGGTGGAAGTACCACAAGCTG‑CCGCACAGATTGTTGACGTACCTGAGAAAAAGGTTTCCACTGAGGACGAAGCACAGACAGAAAGCGGACGCAAGAAAGCTCGTGCTGGTGGTAAGAAATCCTTGAGTGTAGCCCGTAGCTCCGGTGGCGGTATCAATATTTAATCAGGAGGTTATCGTGGAAGACTGCATTGAATGGACCGGAGGTGTCAACTCTAAGGGTTATGGTCGTAAGTGGGTTAATgg                                                                                                         <  1:469891/285‑1 (MQ=255)`  
                                                                                                                                                                                                      `ggTTGCTGGCGGTCTAGCTGGTCTGGCTGGTGGTACTGGTGAAGCACAGATGGTGGAAGTACCACAAGCTG‑CCGCACAGATTGTTGACGTACCTGAGAAAGAGGTTTCCACTGAGGACGAAGCACAGACAGAAAGCGGACGCAAGAAAGCTCGTGCTGGCGGTAAGAAATCCTTGAGTGTAGCACGTAGCTCCGGTGGCGGTATCAACATTTAATCAGGAGGTTATCGTGGCAGACTGCATTGAATGGACCGGAGGTGTCAACTCTAAGGGTTATGGTCGTAAGtctgtctcttatacaca                                                                                                   >  1:211794/1‑285 (MQ=255)`  
                                                                                                                                                                                                            `tGGCGGTCTAGCTGGTCTGGCTGGTGGTACTGGTGAAGCACAGATGGTGGAAGTACCACAAGCTG‑CCGCACAGATTGTTGACGTACCTGAGAAAAAGGTTTCCACTGAGGACGAAGCACAGACAGAAAGCGGACGCAAGACAGCTCGTGCTGGCGGTAAGAAATCCTTGAGTGTAGCCCGTAGCTCCGGTGGCGGTATCAACATTTAATCAGGAGGTTATCGTGGAAGACTGCATTGAATGGACCGGAGGTGTCAACTCTAAGGGTTATGGTCGTAAGTGGGTTAATGGTAAACTTGTGAc                                                                                             <  1:172364/301‑1 (MQ=255)`  
                                                                                                                                                                                                            `tGGCGGTCTAGCTGGTCTGGCTGGGGGTACTGGTGAAGCACAGATGGTGGAAGTACCACAAGCTG‑CCGCACAGATTGTTGACGTACCTGAGAAAAAGGTTTCCACTGAGGACGAAGCACAGACAGAAAGCGGACGCAAGAAAGCTCGTGCTGGCTGTAAGAAATCCTTGAGTGTAGCCCGTAGCTCCGGTGGCGGTATCAACATTTAATCAGGAGGTTATCGTGGAAGACTGCATTGAATGGACCGGAGGTGTCAACTCTAAGGGTTATGGTCGTAAGTGGGTTAATGGTAAACTTGTGAc                                                                                             <  1:678635/301‑1 (MQ=255)`  
                                                                                                                                                                                                              `gCGGTCTAGCTGGTCTGGCTGGTGGTACTGGTGAAGCACAGATGGTGGAAGTACCACAAGCTG‑CCGCACAGATTGTTGACGTACCTGAGAAAAAGGTTTCCACTGAGGACGAAGCACAGACAGAAAGCGGACGCAAGAAAGCTCGTGCTGGCGGTAAGAAATCCTTGAGTGTAGCCCGTAGCTCCGGTGGCGGTATCAACATTTAATCAGGAGGTTATCGTGGAAGACTGCATTGAATGGACCGGAGGTGTCAACTCTAAGGGTTATGGTCGTAAGTGGGTTAATGGTAAACTTGTGACTc                                                                                           <  1:265512/301‑1 (MQ=255)`  
                                                                                                                                                                                                              `gCGGTCTAGCTGGTCTGGCTGGTGGTACTGGTGAAGCACAGATGGTGGAAGTACCACAAGCTG‑CCGCACAGATTGTTGACGTACCTGAGAAAAAGGTTTCCACTGAGGACGAAGCACAGACAGAAAGCGGACGCAAGAAAGCTCGTGCTGGCGGTAAGAAATCCTTGAGTGTAGCCCGTAGCTCCGGTGGCGGTATCAACATTTAATCAGGAGGTTATCGTGGAAGACTGCATTGAATGGACCGGAGGTGTCAACTCTAAGGGTTATGGTCGTAAGTGGGTTAATGGTAAACTTGTGACTc                                                                                           >  1:272404/1‑301 (MQ=255)`  
                                                                                                                                                                                                                    `tAGCTGGTCTGGCTGGTGGTACTGGTGAAGCACAGATGGTGGAAGTACCACAAGCTG‑CCGCACAGATTGTTGACGTACCTGAGAAAAAGGTTTCCACTGAGGACGAAGCACAGACAGAAAGCGGACGCAAGAAAGCTCGTGCTGGCGGTAAGAAATCCTTGAGTGTAGCCCGTAGCTCCGGTGGCGGTATCAACATTTAATCAGGAGGTTATCGTGGAAGACTGCATTGAATGGACCGGAGGTGTCAACTCTAAGGGTTATGGTCGTAAGTGGGTTAATGGTAAACTTGTGACTCCACATa                                                                                     <  1:32400/301‑1 (MQ=255)`  
                                                                                                                                                                                                                    `tAGCTGGTCTGGCTGGTGGTACTGGTGAAGCACAGATGGTGGAAGTACCACAAGCTG‑CCGCACAGATTGTTGACGTACCTGAGAAAAAGGTTTCCACTGAGGACGAAGCACAGACAGAAAGCGGACGCAAGAAAGCTCGTGCTGGCGGTAAGAAATCCTTGAGTGTAGCCCGTAGCTCCGGTGGCGGTATCAACATTTAATCAGGAGGTTATCGTGGAAGACTGCATTGAATGGACCGGAGGTGTCAACTATAAGGGTTATGGTCGTAAGTGGGTTAATGGTAAACTTGTGACTCCACATa                                                                                     <  1:282603/301‑1 (MQ=255)`  
                                                                                                                                                                                                                    `tAGCTGGTCTGGCTGGTGGTACTGGTGAAGCACAGATGGTGGAAGTACCACAAGCTG‑CAGCACAGATTGTTGACGTACCTGAGAAAAAGGTTTCCACTGAGGACGAAGCACAGACAGAAAGCGGACGCAAGAAAGCTCGTGCTGGAGGTAAGAAATCCTTGAGTGTAGCCAGTAGCTCCGGTGGCGGTATCAACATTTAATCAGGAGGTTATCGTGGAAGACTGCATTGAATGGACCGGAGGTGTCAACTCTAAGGGTTATGGTCGTAAGTGGGTTAATGGTAAACTTGTGACTCCACATa                                                                                     <  1:247804/301‑1 (MQ=255)`  
                                                                                                                                                                                                                    `tAGCGGGTCTGGCTTGTGTTACTGGTGAATCACAGATGGTGGAAGTACCAAAAGCTG‑CCGCACAGATTGTTGACGTACCTGAGAAAAAGGTTTCCACTGAGGACGAAGCACAGACAGAAAGCGGACGCAAGAAAGCTCGTGCTTGCGGTAAGAAATCCTTGAGTGTAGCCCGTAGCTCCGGTGGCGGTATTAACATTTAATCAGGAGGTTATCGTGGAAGACTGCATTGAATGGACCGGAGGTGTCAACTCTAAGGGATATGGTCGTAAGTGGGTTAATGGTAAACTTGTGACTCCACATa                                                                                     <  1:148321/301‑1 (MQ=255)`  
                                                                                                                                                                                                                       `gtgtataagagacaggtACTGGTGAAGCACAGATGGTGGAAGTACCACAAGCTG‑CCGCACAGATTGTTGACGTACCTGAGAAAAAGGTTTCCACTGAGGACGAAGCACAGACAGAAAGCGGACGCAAGAAAGCTCGTGCTGGCGGTAAGAAATCCTTGAGTGTAGCCCGTAGCTCCGGTGGCGGTATCAACATTTAATCAGGAGGTTATCGTGGAAGACTGCATTGAATGGACCGGAGGTGTCAACTCTAAGGGTTATGGTCGTAAGTGGGTTAATGGTAAACTTGTGACTCCACATAGGc                                                                                  <  1:61223/287‑1 (MQ=255)`  
                                                                                                                                                                                                                       `cTGGTCTGGCTGGTGGTACTGGTGAAGCACAGATGGTGGAAGTACCACAAGCTG‑CCGCACAGATTGTTGACGTACCTGAGAAAAAGGTTTCCACTGAGGACGAAGCACAGACAGAAAGCGGACGCAAGAAAGCTCGTGCTGGCGGTAAGAAATCCTTGAGTGTAGCCCGTAGCTCCGGTGGCGGTATCAACATTTAATCAGGAGGTTATCGTGGAAGACTACATTGAATGGACCGGAGGTGTCAACTCTAAGGGTTATGGTCGTAAGTGGGTTAATGGTAAACTTGTGACTCCACATAGGc                                                                                  <  1:474921/301‑1 (MQ=255)`  
                                                                                                                                                                                                                       `cTGGTCTGGCTGATGGTACTGGTGAAGCACAGATGGTGGAAGTACCACAAGCTG‑CCACACAGATTGTTGACGTACCTGAGAAAAAGGTTTCCACTGAGGACGAAGCACAGACAGAAAGCGGACGCAAGAAAGCTCGTGCTGGCGGTAAGAAATCCTTGAGTGTAGCCAGTAGCTCCGGTGGCGGTATCAACATTTAATCAGGAGGTTATCGTGGAAGACTGCATTGAATGGACCGGAGGTGACAACTCTAAGGGTTATGGTCGTAAGTGGGTTAATGGTAAACTTGTGACTCCACATAGGc                                                                                  <  1:637409/301‑1 (MQ=255)`  
                                                                                                                                                                                                                         `ggTCTGGCTGGTGGTACTGGTGAAGCACAGATGGTGGAAGTACCACAAGCTG‑CCGCACAGATTGTTGACGTACCTGAGAAAAAGGTTTCCACTGAGGACGAAGCACAGACAGAAAGCGGACGCAAGAAAGCTCGTGCTGGCGATAAGAAATCCTTGAGTGTAGCCCGTAGCTCTGGTGGCGGTATCAACATTTAATCAGGAGGTTATCGTGGAAGACTGCATTGAATGGACCGGAGGTGTCAACTCTAAGGGTTATGGTCGTAAGTGGGTTAATGGTAAACTTATGACTCCACATAGGcac                                                                                >  1:465874/1‑301 (MQ=255)`  
                                                                                                                                                                                                                          `gTCTGGCTGGTGGTACTGGTGAAGCACAGATGGTGTAAGTACCACAAGCTG‑CCGCACAGATTGTTGACGTACCTGAGAAAAAGGTTTCCACTGAGGACGAAGCACAGACAGAAAGCGGACGCAAGAAAGCTCGTGCTGGCGGTAAGAAATCCTTGAGTGTAGCCCGTAGCTCCGGTGGCGGTATCAACATTTAATCATGAGGTTATCGTGGAAGACTGCATTGAATGGACCGGAGGTGTCAACTCTAAGGGTTATGGTCGTAAGTGGGTTAAtctgtctcttatacacctctccgagccca                                                                               >  1:627799/1‑273 (MQ=255)`  
                                                                                                                                                                                                                                 `tggtggTACTGGGGAAGCACAGATGGTGGAAGTACCACAAGCTG‑CCGCACAGATTGTTAACGTACCTGAGAAAAAGGTTTCCACTGAGGACGAAGCACAGACAGAAAGCGGACGCAAGAAAGCTCGTGCTGGCGGTAAGAAATCCTTGAGTGTAGCCCGTAGCTCCGGTGGCGGTATCAACATTTAATCAGGAGGTTATCGTGGAAGACTGCATTGAATGGACCGGAGGTGTCAACTCTAAGGGTTATGGTCGTAAGTGGGTTAATGGTAAACTTGTGACTCCACATAGGCACATCTATga                                                                        <  1:642192/301‑1 (MQ=255)`  
                                                                                                                                                                                                                                  `ggtggtACTGGTGAAGCACAGATGGTGGAAGTACCACAAGCTG‑CCGCACAGATTGTTGACGTACCTGAGAAAAAGGTTTCCACTGAGGACGAAGCACAGACAGAAAGCGGACGCAAGAAAGCTCGTGCTGGCGGTAAGAAATCCTTGAGTGTAGCCCGTAGCTCCTGTGGCGGTATCAACATTTAATCAGGAGGTTATCGTGGAAGACTGCATTGAATGGACCGGAGGTGTCAACTCTAAGGGTTATGGTCGTAAGTGGGTTAATGGTAAACTTGTGACTCCACATAGGCACATCTATgag                                                                       >  1:347432/1‑301 (MQ=255)`  
                                                                                                                                                                                                                                   `gtggtACTGGTGAAGCACAGATGGTGGAAGTACCACAAGCTGCCCGCACAGATTGTTGACGTACCTGAGAAAAAGGTTTCCACTGAGGACGAAGCACAGACAGAAAGCGGACGCAAGAAAGCTCGTGCTGGCGGTAAGAAATCCTTGAGTGTAGCCCGTAGCTCCGGTGGCAGTATCAACATTTAATCAGGAGGTTATCGTGGAAGACTGCATTGAATGGACCGGAGGTGTCAACTCTAAGGGTTATGGTCGTAAGTGGGTTAATGGTAAACTTGTGACTCCACATAGGcctgtctcttat                                                                       >  1:199469/1‑290 (MQ=255)`  
                                                                                                                                                                                                                                   `gtggtACTGGTGAAGCACAGATGGTGGAAGTACCACAAGCTG‑CCGCACAGATTGTTGACGTACCTGAGAAAAAGGTTTCCACTGAGGACGAAGCACAGACAGAAAGCGGACGCAAGAAAGCTCGTGCTGGCGGTAAGAAATCCTTGAGTGTAGCCCGTAGCTCCAGTGGCGGTATCAACATTTAATCAGGAGGTTATCGTGGAAGACTGCATTGAATGGACCGGAGGTGTCAACTCTAAGGGTTATGGTCGTAAGTGGGTTAATGGTAAACTTGTGACTCCACATCGGcctgtctcttata                                                                      >  1:186410/1‑289 (MQ=255)`  
                                                                                                                                                                                                                                   `ggagatgtgtataagagacagtggtggAAGTACCACAAGCTG‑CCGCACAGATTGTTGACGTACCTGAGAAAAAGGTTTCCACTGAGGACGAAGCACAGACAGAAAGCGGACGCAAGAAAGCTCGTGCTGGCGGTAAGAAATCCTTGAGTGTAGCCCGTAGCTCCGGTGGCGGTATCAACATTTAATCAGGAGGTTATCGTGGAAGACTGCATTGAATGGACCGGAGGTGTCAACTCTAAGGGTTATGGTCGTAAGTGGGTTAATGGTAAACTTGTGACTCCACATAGGCACATCTATgagg                                                                      <  1:161695/280‑1 (MQ=255)`  
                                                                                                                                                                                                                                   `ggagaggtgtataagagacagtggtggAAGTACCACAAGCTG‑CCGCACAGATTGTTGACGTACCTGAGAAAAAGGTTTCCACTGAGGACGAAGCACAGACAGAAAGCGGACGCAAGAAAGCTCGTGCTGGCGGTAAGAAATCCTTGAGTGTAGCCCGTAGCTCCGGTGGCGGTATCAACATTTAATCAGGAGGTTATCGTGGAAGACTGCATTGAATGGACCGGAGGTGTCAACTCTAAGGGTTATTGTCGTAAGTGGGTTAATGGTAAACTTGTGACTCCACATAGGCACATCTATgagg                                                                      <  1:381609/280‑1 (MQ=255)`  
                                                                                                                                                                                                                                   `ataagagacaggAAGCAAAGATGGTGGAAGGACCACAAGCTG‑CAGCACAGATTGTTGACGTACCTGAGAAAAAGGTTTCCACTGAGGACGAAGCACAGACAGAAAGCGGACGCAAGAAAGCTCGTGCTGGCGGTAAGAAATCCTTGAGTGTAGCCCGTAGCTCCGGTGGCGGTATCAACATTTAATCAGGAGGTTATCGTGGAAGACTGCATTGAATGGACCGGAGGTGTCAACTCTAAGGGTTATGGTCGTAAGTGGGTTAATGGTAAACTTGTGACTCCACATAGGCACATCTATgagg                                                                      <  1:302463/290‑1 (MQ=255)`  
                                                                                                                                                                                                                                    `tggtACTGGTGAAGCACAGATGGTGGAAGTACCACAAGCTG‑CCGCACAGATTGTTGACGTACCTGAGAAAAAGGTTTCCACTGAGGACGAAGCACAGACAGAAAGCGGACGCAAGAAAGCTCGTGCTGGCGGTAAGAAATCCTTGAGTGTAGCCCGTATCTCCGGTGGCGGTATCAACATTTAATCAGGAGGTTATCGTGGAAGACTGCATTGAATGGACCGGAGGTGTCAACTCTAAGGGTTATGGTCGTAAGTGGGTTAATGGTAAACTTGTGACTCCACATAGGCACATCTATgaggg                                                                     >  1:386788/1‑300 (MQ=255)`  
                                                                                                                                                                                                                                     `ggtACTGGTGAAGCACAGATGGTGGAAGTACCACAAGCTG‑CCGCACAGATTGTTGACGTACCTGAGAAAAAGGTTTCCACTGAGGACGAAGTACAGACAGAAAGCGGACGCAAGAAAGCTCGTGCTGGTGGTAAGAAATCCTTGAGTGTAGCCCGTAGCTCCGGTGGCGGAATCAACATTTAATCCGGAGGTTATCGTGGAAGACTGCATTGAATGGACCGGAGGTGTCAACTCTAAGGGTTATTGTCGTAAGTGGGTTAATTGTAAACTTGTGACTCCACATAGGCACATCTATgaggct                                                                    >  1:528974/1‑299 (MQ=255)`  
                                                                                                                                                                                                                                     `ggtACTGGTGAAGCACAGATGGTGGAAGTACCACAAGCTG‑CCGCACAGATTGTTGACGTACCTGAGAAAAAGGTTTCCACTGAGGACGAAGCACAGACAGAAAGCGGACGCAAGAAAGCTCGTGCTGGCGGTAAGAAATCCTTGAGTGTAGCCCGTAGCTCCGGTGGCGGTATCAACATTTAATCAGGAGGTTTTCGTGGAAGACTGCATTGAATTGACCGGAGGTGTCAACTCTAAGGGTTATGGTCGTAAGTGGGTTAATGGTAAACTTGTGACTCCACATAGGCACATCTATgaggag                                                                    >  1:545964/1‑301 (MQ=255)`  
                                                                                                                                                                                                                                     `ggtACTGGTGAAGCACAGATGGTGGAAGTACCACAAGCTG‑CCGCACAGATTGTTGACGTACCTGAGAAAAAGGTTTCCACTGAGGACGAAGCACAGACAGAAAGCGGACGCAAGAAAGCTCGTGCTGGCGGTAAGAAATCCTTGAGTGTAGCCCGTAGCTCCGGTGGCGGTATCAACATTTAATCAGGAGGTTATCGT‑GAAGACTGCATTGAATGGACCGGAGGTGTCAACTCTAAGGGTTATGGTCGTAAGTGGGTTAATGGTAAACTTGTGACTCCACATAGGCACATCTATGAGgaga                                                                   >  1:310706/1‑301 (MQ=255)`  
                                                                                                                                                                                                                                        `aCTGGTGAAGCACAGATGGGGGAAGTACCACAAGCTG‑CCGAACAGATTGTTGACGTACCTGAGAAAAAGGTTTCCAAGGAGGACGAAGCACAGACAGAAAGCTGACGCAATAAAGCTCGTGCTGGCGGTAAGAAATCCTTGAGTGTAGCCCGTAGCTCCGGTGGCGGTATCAACATTTAATCAGGAGGTTATCGTGGAAGACTGCATTGAATGGACCGGAGGTGTCAACTCTAAGGGTTATGGTCGTAAGTGGGTTAATGGTAAACTTGTGACTCCACATAGGCACATCTATGAGGAGACa                                                                 <  1:596380/301‑1 (MQ=255)`  
                                                                                                                                                                                                                                           `ggTGAAGCACAGATGGTGGAAGTACCACAAGCTG‑CCGCACAGATTGTTGACGTACCTGAGAAAAAGGTTTCCACTGAGGACGAAGCACAGACAGAAAGCGGACGCAATAAAGCTCGTGCTGGCGGTAAGAAATCCTTGAGTGTAGCCCGTAGCTCCGGTGGCGGTATCAACATTTAATCAGGAGGTTATCGTGGACGACTGCATTGAATGGACCGGAGGTGTCAACTCTAAGGGTTATGGTCGTAAGTGGGTTAATGGTAAACTTGTGACTCCACATAGGCACATCTATTAGGAGACatat                                                              >  1:631395/1‑301 (MQ=255)`  
                                                                                                                                                                                                                                             `tGAAGCACAGATGGTGGAAGTACCACAAGCTG‑CCGCACAGATTGTTGACGTACCTGAGAAAAAGGTTTCCACTGAGGACGAAGCACAGACAGAAAGCGGACGCAAGAAAGCTCGTGCTGGCGGTAAGAAATCCTTGAGTGTAGCCCGTAGCTCCGGTGGCGGTATCAACATTTAATCAGGAGGTTATCGTGGAAGACTGAATTGAATGGACCGGAGGTGTCAACTCTAAGGGTTATGGTCGTAAGTGGGTTAATGGTAAACTTGTGACTACACATAGGCACATCTATGAGGAGACATATgg                                                            <  1:289490/301‑1 (MQ=255)`  
                                                                                                                                                                                                                                                `aGCACAGATGGTGGAAGTACCATAAGCTG‑CCGCACAGATTGTTGACGTACCTGAGAAAAAGGTTTCCACTGAGGACGAAGCACAGACAGAAAGCGGACGCAAGAAAGCTCGTGCTTGCGGTAAGAAATCCTTGAGTGTAGCCCGTAGCTCCGGTGGCGGTATCAACATTTAATCAGGAGGTTATCGTGGAAGACTGCATTGAATGGACCGGAGGTGTCAACTCTAAGGGTTATGGTCGTAAGTGGGTTAATGGTAAACTTGTGACTCCACATAGGCACATCTATGAGGAGACATATGGTcc                                                         >  1:226907/1‑301 (MQ=255)`  
                                                                                                                                                                                                                                                    `caGATGGTGGAAGTACCACAAGCTG‑CAGCACAGATTGTTGACGTACCTGAGAAAAAGGTTTCCACTGAGGACGAAGCACAGACAGAAAGCGGACGCAAGAAAGCTCGTGCTGGCGGTAAGAAATCCTTGAGTGTAGCCCGTAGCTCCGGTGGAGGTATCAACATTTAATCAGGAGGTTATCGTGGAAGACTGCATTGAATGGACCGGAGGTGTCAACTCTAAGGGTTATGGTCGTAAGTGGGTTAATGGTAAACTTGTGACTCCACATAGGCACATCTATGAGGAGACATATGGTccctgt                                                     >  1:575112/1‑297 (MQ=255)`  
                                                                                                                                                                                                                                                     `ggATGGTGGAAGTACCACAAGCTG‑CCGCACAGATTGTTGACGTACCTGAGAAAAAGGTTTCCACTGAGGACGAAGCACAGACAGAAAGCGGACGCAAGAAAGCTCGTGCTGGCGGTAAGAAATCCTTGAGTGTAGCCCGTAGCTCCGGTGGCGGTATCAACATTTAATCAGGAGGTTATCGTGGAAGACTGCATTGAATGGACCGGAGGTGTCAACTCTAAGGGTTATGGTCGTAAGTGGGTTAATGGTAAACTTGTGACTCCACATAGGCACATCTATGAGGAGACATATGGTCCAGTTc                                                    <  1:721724/300‑1 (MQ=255)`  
                                                                                                                                                                                                                                                      `gATGGTGGAAGTACCACAAGCTG‑CCGCACACATTGTTGACGTACCTGAGAAAATGGTTTCCACTGCGGACGACGCACAGACAGAACGCGGACGCAAGAAAGCTCGTGCTGGCGGTAAGACATCCTTGAGTGTAGCCCGTAGCTCCGGTGGCGGTATCAACCTTTAATCAGGAGGTTATCGTGGACGACTGCATTGAATGGACCGGAGGTGTCAACTCTCAGCGTTATGGTCGTAAGTTGGTTAATGGTAAACTTGTGACTCCACATAGGCACATCTATTAGGAGACATATGGTCCAGTTcc                                                   >  1:540461/1‑301 (MQ=255)`  
                                                                                                                                                                                                                                                          `gtggAAGTACCACAAGCTG‑CCGCACAGATTGTTGACGTACCTGAGAAAAATGTTTCCACTGAGGACGAAGCACAGACAGAAAGCGGACGCAAGAAAGCTCGTGCTGGCGGTAAGAAATCCTTGAGTGTAGCCCGTAGCTCCGGTGGCGGTATCAACATTTAATCAGGAGGTTATCGTGGAAGACTGCATTGAATGGACCTGAGGTGTCAACTCTAAGGGTTATGGTCGTAAGTGGGTTAATGGTAAACTTGTGACTCCACATCGGCACATCTATTAGGAGACATATGGTCCAGTTCCAACa                                               >  1:4005/1‑301 (MQ=255)`  
                                                                                                                                                                                                                                                          `gtggAAGTACCACAAGCTG‑CCGCACAGATTGTTGACGTACCTGAGAAAAAGGTTTCCACTGAGGACGAAGCACAGACAGAAAGCAGACGCAAGAAAGCTCGTGCTGGCGGTAAGAAATCCTTGAGTGTAGCCCGTAGCTCCGGTGGCGGTATCAACATTTAATCAGGAGGTTATCGTGGAAGACTGCATTGAATGGACCGGAGGTGTCAACTCTAAGGGTTATGGTCGTAAGTGGGTTAATGGTAAACTTGTGACTCCACATAGGCACATCTATGAGGAGACatactgtctcttatacaca                                               >  1:274686/1‑285 (MQ=255)`  
                                                                                                                                                                                                                                                           `tggAAGTACCACAAGCTG‑CCGAACAGATTGTTGACGTACCTGAGAAAAAGGTTTCCACTGAGGACGAAGCACAGACAGAAAGCGGACGCAAGAAAGCTCGTGCTGGCGGTAAGAAATCCTTGAGTGTAGCCCGTAGCTCCGGTGGCGGTATCAACATTTAATCAGGAGGTTATCGTGGAAGACTGCATTGAATGGACCGGAGGTGTCAACTCTAAGGGTTATGGTCGTAAGTGGGTTAATGGTAAACTTGTGACTCCACATAGGCACATCTATGAGGAGACATATGGTCCAGTTCCAACAg                                              <  1:75616/301‑1 (MQ=255)`  
                                                                                                                                                                                                                                                                          `cTG‑CCGAACAGATTGTTGACGTACCTGAGAAAGAGGTTTCCACTGAGGACGAAGCACAGACAGAAAGCGGACGCAAGAAAGCTCGTGCTGGCGGTAAGAAATCCTTGAGTGTAGCCCGTAGCTCCGGTGGCGGTATCAACATTTAATCAGGAGGTTATCGTGGAAGACTGCATTGAATGGACCGGAGGTGTCAACTCTAAGGGTTATGGTCGTAAGTGGGTTAATGGTAAACTTGTGACTCCACATAGGCACATCTATTAGGAGACATATGGTCCAGTTCCAACAGGAATTGTGGTGATGc                               <  1:317507/301‑1 (MQ=255)`  
                                                                                                                                                                                                                                                                            `g‑CCGCACAGATTGTTGACGTACCTGAGAAAAAGGTTTCCACTGAGGACGAAGCACAGACAGAAAGCGGACGCAAGAAAGCTCGTGCTGGCGGTAAGAAATCCTTGAGTGTAGCCCGTAGCTCCGGTGGCGGTATCAACATTTAATCAGGAGGTTATCGTGGAAGACTGCATTGAATGGACCGGAGGTGTCAACTCTAAGGGTTATGGTCGTAAGTGGGTTAATGGTAAACTTGTGACTCCACATAGGCACATCTATGAGGAGACATATGGTCCAGTTCCAACAGGAATTGTGGTGATGCat                             >  1:656846/1‑301 (MQ=255)`  
                                                                                                                                                                                                                                                                                     `gATTGTTGACGTACCTTAGAAAAAGGTTTCAACTGAGGACGAAGCACAGACAGAAAGCGGACGCAAGAAAGCTCGTGCTGGCGGTAAGAAATCATTGAGTGTAGCCCGTAGCTCCGGTGGCGGTATCAACATTTAATCAGGAGGTTATCGTGGAAGACTGCATTGAATGGACCGGAGGTGTCAACTAGAAGGGTTATGGGCGTAAGTGGGTTAATGGTAAACTTGTGAATCCACATAGGCACATCTATGAGGAGACAAATGATCCAGGTCCAACAGAAATTGTGGTGAGGctaaactgaga                     >  1:128644/1‑291 (MQ=255)`  
                                                                                                                                                                                                                                                                                     `gATTGTTGACGTACCTGAGAAAAAGGTTTCCACTGATGACGAAGCACAGACAGAAAGCGGACGCAAGAAAGCTCGTGCTGGCGGTAAGAAATCCTTGAGTGTAGCCCGTAGCTCCGGTGGCGGTATCAACATTTAATCAGGAGGGTATCGTGGAAGAATGCATTGAATGGACCGGAGGTGTCAACTCTAAGGGTTATGGTCGTAAGTGGGTTAATGGCAACCTTGTGACTCCACATAGGCACATCTATGAGGAGACATCTGGTCCAGTTCCAACAGGAATTCTGGTGATGCATATCTGCGa                     >  1:360281/1‑301 (MQ=255)`  
                                                                                                                                                                                                                                                                                       `aagttgACGTACCTGAGAAAAAGGTGTCCACTGAGGACGAAGCACAGACAGAGAGCGGACGCAAGAAAGCTCGTGTTAGCGGTAAGAAAGCCTTGAGTGTAGCCAGTAGCTCCGGTGGCGGTATCAACATTTAATCAGGAGGTTATCGTGGAATACTGCATTGAATGGACTGGAGGTGTCAACTCTAAGGGTTATGGTCGTAAGTGGGTTAATGGTAAACTTGTGACTCCACATAGGCACATCTATGAGGAGACATATGGTCCAGTTCCAACAGGAATTGTGGTGATGCATATCTGCGATa                   <  1:446997/299‑1 (MQ=255)`  
                                                                                                                                                                                                                                                                                         `gttgACGTACCTGAGAAAAAGGTTTCCACTGAGGACGAAGCACAGACAGAAAGCGGACGCAAGCAAGCTCGTGCTGGCCGTAATAAATACTTGAGTGTAGCCCGTAGCTCCGGTGGCGGTATCAACATTTCATCAGGAGGTTATCGTGGAAGACTGCATTGAATGGACAGGAGTTGTCAACTCTAAGGGTTATGGTCGTAAGTGGGTTAATGGTACACTTTTGACTCCACATAGGCACATCTATGAGGAGCCATATGGTCCCGTTCCAACAGGCATTGTGGTGATGCATATCTGCGATAAc                 >  1:259276/1‑301 (MQ=255)`  
                                                                                                                                                                                                                                                                                         `gttgACGTACCTGAGAAAAAGGTTTCCACTGAGGACGAAGCACAGACAGAAAGCGGACGCAAGAAAGCTCGTGCTGGCGGTAAGAAATCCTTGAGTGTAGCCCGTAGCTCCGGTGGCGGTATCAACATTTAATCAGGAGGTTATCGTGGAAGACTGCATTGAATGGACCGGAGGTGTCAACTCTAAGGGTTATGGTCGTAAGTGGGTTAATGGTAAACTTGTGACTCCACATAGGCACATCTATGAGGAGACATATGGTCCAGTTCCAACAGGAATTGTGGTGATGCAGATCTGCGATAAc                 >  1:702777/1‑301 (MQ=255)`  
                                                                                                                                                                                                                                                                                               `gTACCTGAGAAAAAGGTTTCCACTGAGGACGAAGCACAGACAGAAAGCGGACGCAAGAAAGCTCGTGCTGGCGGTAAGAAATCCTTGAGTGTAGCCCGTAGCTCCGGTGGCGGTATCAACATTTAATCAGGAGGTTATCGTGGAAGACTGCATTGAATGTACCGGAGGTGTCAACTCTAAGGGTTATGGTCGTAAGTGGGTTAATGGTAAACTTGTGACTCCACATAGGCACATCTATAAGGAGACATATGGTCCAGTTCCGACAGGAATTGTGGTGATGCATATCTGCGATAACCCTAgg           <  1:441799/301‑1 (MQ=255)`  
                                                                                                                                                                                                                                                                                               `gTACCTGAGAAAAAGGTTTCCACGGAGGACGAAGCACAGACAGAAAGCGGACGCAAGAAAGCTCGTGCTGGCGGTAAGAAATCCTTGAGTGTAGCCCGTAGCTCCGGTGGCGGTATCAACATTTAATCAGGAGGTTATCGTGGAAGACTGCATTGAATGGACCGGAGGTGTCAACTCTAAGGGTTATGGTCGTAAGTGGGTTAATGGTAAACTTGTGACTCCACATAGGCACATCTATGAGGAGACATAAGGTACAGTTCCAACAGGAATTGTGGTGATGCATATCTGCGATAACCCTAgg           <  1:412284/301‑1 (MQ=255)`  
                                                                                                                                                                                                                                                                                                `aaCCTGAGAAAAAGGTTTCCACTGAGGACGAAGCACAGACAGAAAGCGGACGCAAGAAAGCTCGGGCTGGCGGTAAGAAATCCTTGAGTGTAGCCCGTAGCTCCGGTGGCGGTATCAACATTTAATCAGGAGGTTATCGTGGAAGACTGCATTGAATGGACCGGAGGTGTCAACTCTAAGGGTTATGGTCGTAAGTGGGTTAATGGTAAACTTGTGACTCCACATAGGCACATCTATGAGGAGACATATGGTCCAGTTCCAACAGGAATTGTGGTGATGCATATCTGCGATAACCCTAGGt          <  1:744031/300‑1 (MQ=255)`  
                                                                                                                                                                                                                                                                                                  `ccTGAGAAAAAGGTTTCCACTGAGGACGAAGCACAGACAGAAAGCGGACGCAAGAAAGCTCGTGCTGGCGGTAAGAAATCCTTGAGTGTAGCCCGTAGCTCCGGTGTCGGTATCAACATTTAATCAGGAGGTTATCGTGGAAGACTGCATTGAATGGACCGGCGGTGTCAACTCTAAGGGTTATGGTCGTAAGTGGGTTAATGGTAAACTTGTGACTCCACATAGGCACATCTATGAGGAGACAACTGGTACAGTTCCAACAGGAATTGTGGTGATGAATATCTGCGATAACCCTAGGTGc        >  1:134075/1‑301 (MQ=255)`  
                                                                                                                                                                                                                                                                                                     `gagaAAAAGGTTTCCACTGAGGACGAAGCACAGACAGCAAGCGGACGCAAGAAAGCTAGTGCTGGCGGTAAGAAATCCTTGAGTGTAGCCCGTAGCTCCGGTGGCGGTATCAACATTTAATCAGGAGGTTATCGTGGAAGACTGCATTGAATGGACCGGAGGTGTCAACTCTAAGGGTTATGGTCGTAAGTGGGTTAATGGTAAACTTGTGACTCCACATAGGCACATCTATGAGGAGACATATGGTCCAGTTCCAACAGGAATTGTGGTGATGCATATCTGctgtctcttatacacatct     >  1:740315/1‑283 (MQ=255)`  
                                                                                                                                                                                                                                                                                                     `gagaAAAAGGTTTCCACTGAGGACGAAGCACAGACAGAAAGCGTACGCAAGAAAGCTCGTGCTGGCGGTAAGAAATCCTTGAGTGTAGCCCGTAGCTCCGGTGGCGGTATCAACATTTAATCAGGAGGTTATCGTGGAAGACTGCATTGAATGTACCGGAGGTGTCAACTCTAAGGGTTATTGTCGTAAGTGGGTTAATGGTAAACTTGTGACTCCACATAGGCACATCTATGAGGAGACATATGGTCCAGTTCCAACAGGAATTGTGGTGATGCATATCTGCGATAACACTAGGTGcctg     >  1:714054/1‑298 (MQ=255)`  
                                                                                                                                                                                                                                                                                                     `gagaAAAAGGTTTCCACTGAGGACGAAGCACAGACAGAAAGCGGACGCAAGAAAGCTCGTGCTGGCGGTAAGAAATCCTTGAGTGTAGCCCGTAGCTCCGGTGGCGGTATCAACATTTAATCAGGATGTTATCGTGGAAGACTGCATTGAATGGACCGGAGGTGTCAACTCTAAGGGTTATGGTCGTAAGTGGGTTAATGGTAAACTTGTGACTCCACATAGGCACATCTATGAGGAGACATATGGTCCAGTTCCAACAGGAATTGTGGTGATGCATATCTGCGATAACCCTAGGTGCtat     >  1:792561/1‑301 (MQ=255)`  
                                                                                                                                                                                                                                                                                                       `aaaaaAGGTTTCCACTGAGTACGAAGCACAGACAGAAAGCGGACGCAAGAAAGCTCGTGCTGGCGGTAAGAAATCCTTGAGTGTAGCCCGTAGCTCCGGTGGCGGTATCAACATTTAATCAGGAGGTTATCGTGGAAGACTGCATTGAATGGACCGGTGGTGTCAACTCTAAGGGTTATGCTCGTAAGTGGGTTAATGGTAAACTTGTGACTCCACATAGGCACATCTATGAGGAGACATATGGTCCAGTTCCAACAGGAATTGTGGTGATGCATATCTGCGATAACCCTAGGTGCTATaa   <  1:395581/300‑1 (MQ=255)`  

                                                                                                                                                                                                                                                                                                           `|`                                                                                                                                                                                                                                                                                                           
`ATTCCTAGCGAAACGCTTTAAGTACCTTGCGGAACTCCACGATTGCGATTGGGTGTCTCGTTGTAAGCATGAAGGCGAGACAATGCGTGTATACTTTAAGGAGGTATAAGTTATGGGTAAGAAAGTTAAGAAGGCCGTGAAGAAAGTCACCAAGTCCGTTAAGAAAGTCGTTAAGGAAGGGGCTCGTCCGGTTAAACAGGTTGCTGGCGGTCTAGCTGGTCTGGCTGGTGGTACTGGTGAAGCACAGATGGTGGAAGTACCACAAGCTG‑CCGCACAGATTGTTGACGTACCTGAGAAAGAGGTTTCCACTGAGGACGAAGCACAGACAGAAAGCGGACGCAAGAAAGCTCGTGCTGGCGGTAAGAAATCCTTGAGTGTAGCCCGTAGCTCCGGTGGCGGTATCAACATTTAATCAGGAGGTTATCGTGGAAGACTGCATTGAATGGACCGGAGGTGTCAACTCTAAGGGTTATGGTCGTAAGTGGGTTAATGGTAAACTTGTGACTCCACATAGGCACATCTATGAGGAGACATATGGTCCAGTTCCAACAGGAATTGTGGTGATGCATATCTGCGATAACCCTAGGTGCTATAAC  >  NC_001604.1/19423‑20018`

  

Alignment Legend

Aligned base mismatch/match (shaded by quality score): `ATCG/ATCG` `< 3 ≤` `ATCG/ATCG` `< 11 ≤` `ATCG/ATCG` `< 26 ≤` `ATCG/ATCG` `< 37 ≤` `ATCG/ATCG` `< 38 ≤` `ATCG/ATCG`

Unaligned base: `atcg`    Masked matching base: `atcg`    Alignment gap: `‑`    Deleted base: `‑`
